# Charge transfer from and to manganese phthalocyanine: bulk materials and interfaces

**DOI:** 10.3762/bjnano.8.160

**Published:** 2017-08-04

**Authors:** Florian Rückerl, Daniel Waas, Bernd Büchner, Martin Knupfer, Dietrich R T Zahn, Francisc Haidu, Torsten Hahn, Jens Kortus

**Affiliations:** 1 IFW Dresden, Helmholtzstr. 20, D-01069 Dresden, Germany; 2 Semiconductor Physics, Chemnitz University of Technology, D-09107 Chemnitz, Germany; 3 Institute of Theoretical Physics, TU Bergakademie Freiberg, Leipziger Str. 23, D-09596 Freiberg, Germany

**Keywords:** charge transfer, electronic properties, manganese phthalocyanine

## Abstract

Manganese phthalocyanine (MnPc) is a member of the family of transition-metal phthalocyanines, which combines interesting electronic behavior in the fields of organic and molecular electronics with local magnetic moments. MnPc is characterized by hybrid states between the Mn 3d orbitals and the π orbitals of the ligand very close to the Fermi level. This causes particular physical properties, different from those of the other phthalocyanines, such as a rather small ionization potential, a small band gap and a large electron affinity. These can be exploited to prepare particular compounds and interfaces with appropriate partners, which are characterized by a charge transfer from or to MnPc. We summarize recent spectroscopic and theoretical results that have been achieved in this regard.

## Review

### Introduction

The family of metal-centered phthalocyanines has been considered for future technological applications because of their favorable electronic and optical properties and their advantageous chemical stability [1–8]. Phthalocyanine molecules can harbor a number of metal ions, in particular transition-metal ions such as cobalt, iron or manganese. A special characteristic of transition-metal centered phthalocyanines is, that transition-metal ions often are characterized by a magnetic moment, and therefore such phthalocyanines also show very interesting magnetic behavior [9]. They have even been discussed in terms of molecular magnets including their discussion in future applications in the field of molecular spintronics [10–12].

**Figure 1:**
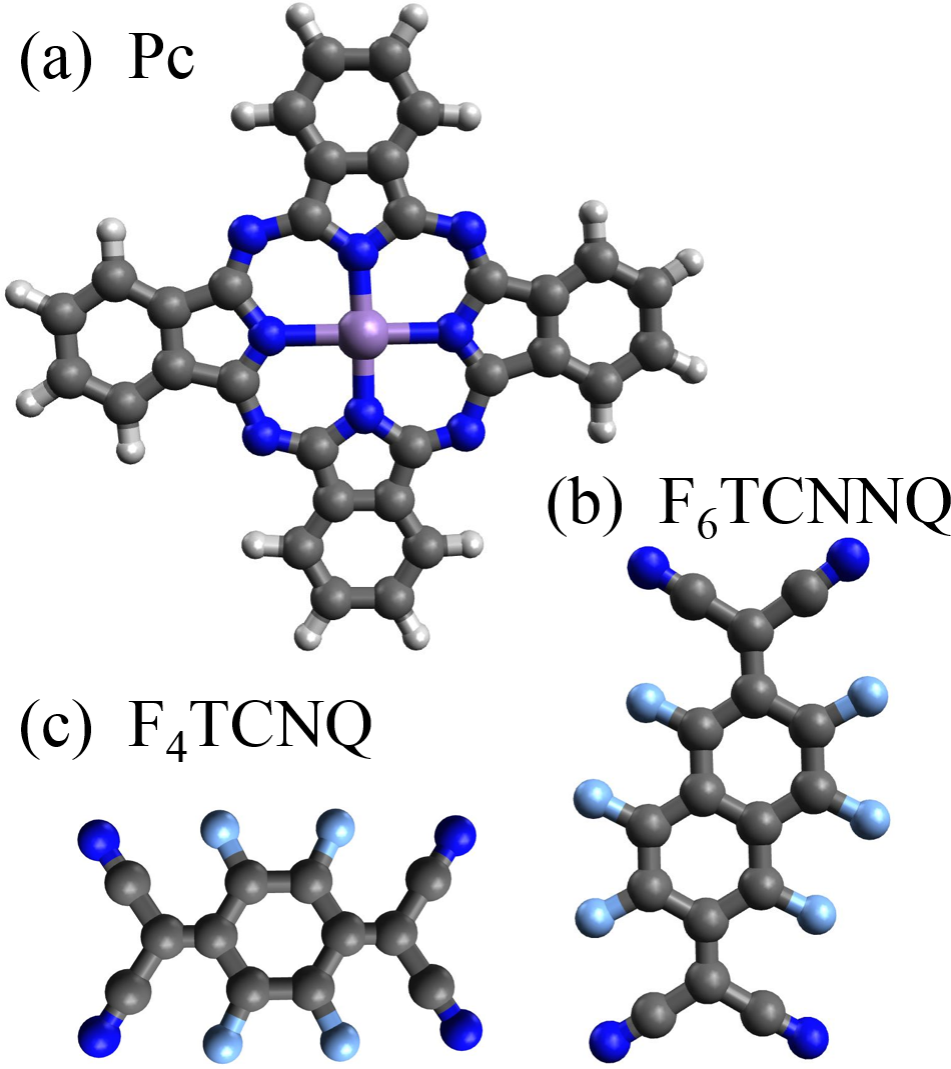
Molecular structure of (a) transition metal phthalocyanines, (b) 1,3,4,5,7,8-hexafluorotetracyanonaphthoquinodimethane (F_6_TCNNQ), and (c) 2,3,5,6-tetrafluoro-7,7,8,8-tetracyanoquinodimethane (F_4_TCNQ). The transition-metal center in the phthalocyanine molecule can vary; in this work Mn and Co are relevant. Moreover, phthalocyanines can be modified by the substitution of hydrogen with fluorine atoms as in F_16_CoPc. Different atoms are shown with different colors (C: black, N: blue, H: white, F: light blue, and Mn/Co: purple).

Among these transition-metal phthalocyanines, manganese phthalocyanine (MnPc) is one of the most interesting molecules due to its particular electronic and magnetic properties in the bulk [13–16]. A schematic representation of the molecule structure is depicted in Figure 1 below. For instance, MnPc is characterized by an unusual *S* = 3/2 spin state of the central Mn(II) ion. The spin of MnPc is a consequence of three unpaired 3d electrons in the Mn 3d levels, which also lie close to the chemical potential. In essence, the electronic properties (partly) reflect the behavior of these 3d electrons, and MnPc plays a special role in the group of the transition-metal phthalocyanines. The energy gap between the occupied and unoccupied molecular orbitals of MnPc is the smallest among all transition-metal phthalocyanines [17–21], its ionization potential also is the smallest within this class of material [17–18,22], while the electron affinity is larger than those of the others [17–18]. Furthermore, the exciton binding energy related to the lowest electronic singlet excitation is somewhat larger compared to, e.g., CuPc [18–20,23]. In Table 1 we summarize these values in comparison to CuPc, the most prominent and most extensively investigated transition-metal phthalocyanine to date.

**Table 1:** Summary of characteristic electronic parameters for MnPc determined for thin films in comparison to those for CuPc. In detail, we compare the ionization potential (IP), the electron affinity (EA), the energy gap as seen in optical studies (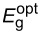), the transport energy gap (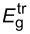), and the exciton binding energy (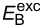) of the lowest singlet excitation (see text for references). All values are given in eV.

	IP	EA	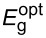	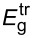	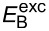

MnPc	4.5	3.3	0.5	1.2	0.7
CuPc	5.0	2.7	1.8	2.3	0.5

These exceptional properties of MnPc render it possible that this molecule can undergo charge-transfer reactions of either kind, i.e., it can be oxidized or reduced by suitable reaction partners. This can be utilized to synthesize new compounds with potentially interesting properties. In this contribution we present a summary of recent results in regard of charge transfer compounds, or interfaces characterized by charge transfer, which all are based on MnPc.

### Materials and methodology

This article covers charge-transfer reactions of manganese phthalocyanine with the alkali metal potassium as well as with further organic molecules. The latter are characterized by a rather large electron affinity to enable charge transfer from MnPc to these structures. In Figure 1 we show the structure of all molecules discussed below. These are MnPc, its phthalocyanine relative F_16_CoPc (both purchased from Sigma-Aldrich), F_4_TCNQ (2,3,5,6-tetrafluoro-7,7,8,8-tetracyanoquinodimethane, TCI Europe) and F_6_TCNNQ (1,3,4,5,7,8-hexafluorotetracyanonaphthoquinodimethane, Novaled AG). F_4_TCNQ is quite well known for its high electron affinity [24], and it is also known to form many charge-transfer crystals with appropriate partners [25–26]. Further, it has also been used as dopant material for organic electronic devices [27–29]. More recently, F_6_TCNNQ has been introduced into organic devices with advantages such as an even higher electron affinity and a larger molecular mass, which prevents diffusion of the molecule in organic devices [30]. F_16_CoPc has been applied recently in a few cases only, in order to induce charge transfer across interfaces to other insulating (or semiconducting) materials [31–33]. The electron affinities of the three acceptor molecules are 4.5 eV (F_16_CoPc [34]), 5.2 eV (F_4_TCNQ [35]) and 5.6 eV (F_6_TCNNQ [36]).

The results presented in this contribution were achieved by either solid-state spectroscopy methods or density functional based calculations. The experimental methods comprise photoelectron (or photoemission) spectroscopy (PES), inverse photoemission spectroscopy (IPES), electron energy-loss spectroscopy (EELS), spectroscopic ellipsometry and X-ray absorption spectroscopy (XAS). Here, we only briefly mention the kind of information that is provided by these methods, and we refer the reader to comprehensive literature for detailed information.

PES [37–39] is based on the photoelectric effect and provides insight into the valence-band electronic density of states as well as the binding energy and line shape of core levels, which give information about the composition of the sample and the chemical state (e.g., valency) of the atoms or ions. In IPES [19,40–42], the unoccupied density of states is probed. EELS [43–45] can also be called inelastic electron scattering and measures the electronic excitations either in the valence-band region, or from core levels into unoccupied states, whereas momentum-dependent studies are possible [43,45–46]. The EELS cross section is proportional to Im(−1/ε) (ε is the dielectric function). In this way, one can investigate valence-band excitations (cf. optical methods) and the element-projected unoccupied density of states. Also, access to orbital selective occupations and the magnetic moment of open shells is accessible. Spectroscopic ellipsometry [47–49] measures the change in the light polarization after reflection on a sample surface. This information allows for the determination of the real and the imaginary part of the dielectric function. XAS [42,50] is equivalent to EELS in the core-level region, and polarization-dependent studies have often been carried out to study the molecular orientation on substrates. In addition to our experiments we performed calculations within the density functional theory (DFT) framework. We used a recent version of the NRLMOL all-electron DFT code [51–52], which uses large Gaussian-orbital basis sets for the representation of the electronic wavefunctions [53]. Unless noted otherwise we used the PBE functional [54] within the general gradient approximation (GGA) was used for all calculations. We semi-empirically included dispersion correction according to the Grimme DFT-D3 method [55] in all of our calculations.

### K*_x_*MnPc: formation of stable phases with MnPc anions

**Figure 2:**
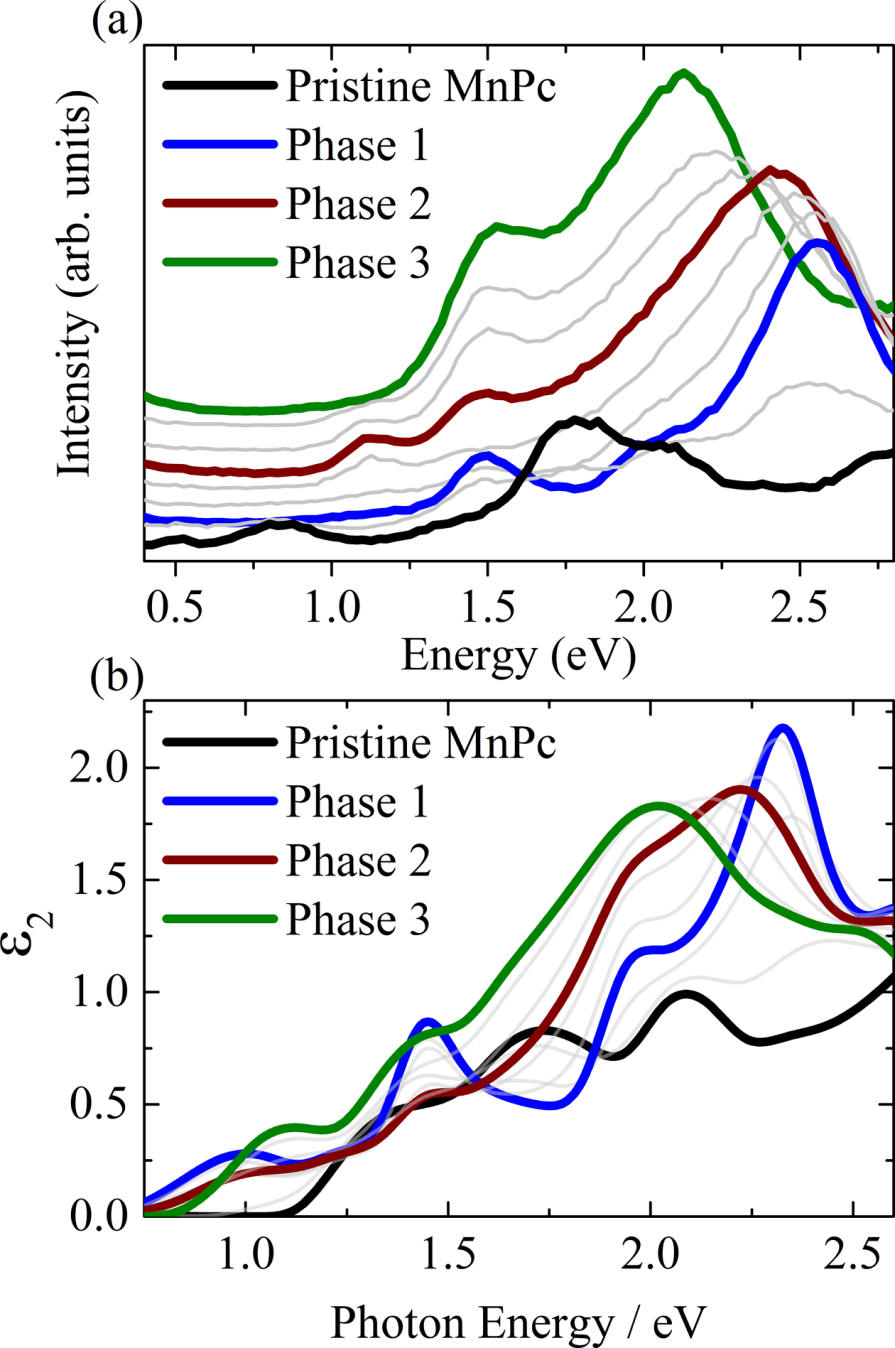
Evolution of the electronic-excitation spectra of MnPc upon potassium doping as determined using electron energy-loss spectroscopy (panel (a)) and spectroscopic ellipsometry (panel (b)). Panel (a) shows the so-called loss function, Im(−1/ε) [56], while panel (b) shows the imaginary part of the dielectric function, ε_2_ [49]. The potassium concentration increases from MnPc over phase 1 to phase 2 and 3. Thicker lines depict the spectra for particular doped phases as described in the text.

The formation of compounds with composition K*_x_*MnPc was achieved by evaporation of potassium from so-called K dispensers (SAES Getters, S.P.A, Italy) onto MnPc thin films under ultra-high vacuum conditions. These then were thoroughly characterized by the spectroscopic methods in order to obtain a comprehensive picture. We start the presentation of our spectroscopic data with the development of the electronic excitation spectra of MnPc as a function of potassium doping. These were determined using EELS and spectroscopic ellipsometry. In Figure 2 we show the corresponding results.

The EELS measurements were carried out at a momentum transfer of 0.1 Å^−1^ which represents the so-called optical limit, i.e., the data are equivalent to those from corresponding optical studies [43]. Note that the ellipsometry results in Figure 2b start at higher energies due to instrumental limitations. The spectrum of pristine MnPc is characterized by several spectral features at about 0.5, 0.8, 1.4, 1.8, and 2.1 eV [23], which are clearly seen in Figure 2a. Around 2 eV, the excitations are usually ascribed to the Q band common to many phthalocyanines [57–59]. They are due to transitions from the highest occupied molecular orbital (HOMO) to the lowest unoccupied molecular orbital (LUMO) [58]. The observed fine structure was attributed to a combination of Davydov splitting, the admixture of charge-transfer excitations as well as vibronic satellites [60–68]. The appearance of further excitations at lower energies for MnPc is still not fully understood. Certainly, they are connected to the contribution of Mn 3d states to the molecular orbitals close to the chemical potential, a complete picture, however, is elusive [23,69–73].

The addition of potassium induces particular changes in regard of the electronic excitations. The two lowest-lying features (at about 0.5 and 0.8 eV, see panel (a)) disappear, also the intensity in the Q band region is drastically reduced. Instead, spectral structures show up at 1.5 eV and about 2.6 eV up to a particular doping level (called phase 1 in Figure 2). Further potassium doping results in the appearance of an excitation at about 1.1 eV, while the structure at about 2.6 eV shows a downshift in energy. The intensity of the excitation at 1.1 eV as seen in panel (a) reaches a maximum at a second distinct doping level (phase 2), thereafter it starts to vanish again. In contrast, the excitation at 1.5 eV is steadily growing in intensity and the highest-lying excitation continues to downshift until a third composition is reached (phase 3).

**Figure 3:**
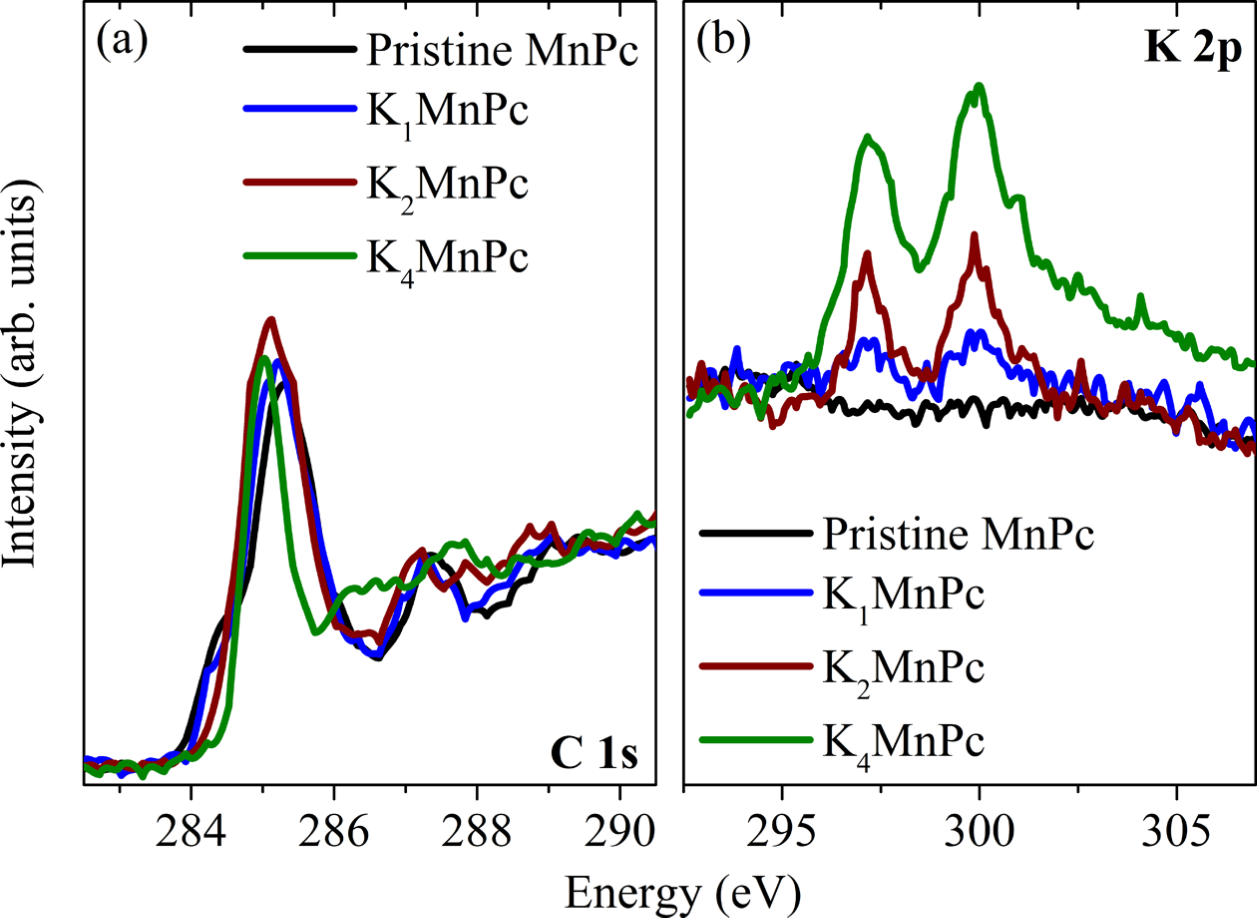
C 1s (panel (a)) and K 2p (panel (b)) excitation edges of MnPc and the three potassium-doped phases (adapted from [56]). The relative intensities of these two edges were used to analyze the composition of the doped phases.

A detailed analysis of these data together with those from electron diffraction [56] revealed the existence of particular K*_x_*MnPc compositions (phases 1, 2, and 3). This, in general, parallels the behavior of other transition-metal phthalocyanines upon potassium doping, where also particular stable phases were reported [74–76]. We emphasize that this conclusion is nicely supported by the fact that all EELS spectra at doping levels between these three phases can be modeled by a corresponding superposition of the spectra of the phases in the direct neighborhood [56]. The exact composition of these phases was finally determined by an analysis of the respective C 1s and K 2p core-level excitations. These data are depicted in Figure 3.

In this Figure, excitation from the C 1s core level in unoccupied π-derived states start at about 285 eV, those into σ-derived carbon states at about 292 eV, while K 2p to K 3d excitations are seen at 297.1 and 298.8 eV [50,77]. The spectral evolution in Figure 3 clearly signals the increasing K content in our samples. The analysis of the relative spectral weights allowed for the determination of the exact composition of the three potassium-doped MnPc phases: K_1_MnPc, K_2_MnPc, and K_4_MnPc [56]. Thus, the spectra highlighted in Figure 2 above represent the electronic excitation spectra of these three phases. In addition, the spectral shape of the C 1s excitation data change in a characteristic manner as a function of doping, in particular right at the excitation onset.

In the case of undoped MnPc, the excitation edge starts with a low-energy shoulder around 284.5 eV before the first maximum at 285.3 eV is reached. Also for other transition-metal phthalocyanines such a C 1s excitation edge is observed [78–80]. This two-peak structure has its origin in the fact that the phthalocyanine ring consists of carbon atoms with different chemical environments, namely those with nitrogen as neighbors and those without. This is clearly seen in corresponding C 1s core-level photoemission data [74,81–82], in which the binding energy for the two carbon species is different. Considering the C 1s excitations as seen in Figure 3a, the excitations from these two carbon sites into the lowest unoccupied orbital give rise to a two-peak structure. Upon doping, there are two changes that impact the C 1s excitations. First, the unoccupied levels are filled with the doping-induced electrons. Second, the carbon binding energies change, as revealed by photoemission data, which show a broadening and the appearance of significantly less structured C 1s core level features in the doped compounds [74,79–80]. As a consequence, the low-energy shoulder in the C 1s excitations is lost and only a single low-energy feature is seen for higher doping levels. Again, this parallels the observations for other doped phthalocyanines [78–80]. There is, however, an important difference to the evolution of the C 1s excitation edges of FePc, CoPc and CuPc upon potassium doping. For these three materials the low-energy shoulder virtually disappeared at a doping level of about one K atom per molecule [78–79,83]. In the case of MnPc here (see Figure 3), this state is reached not until the composition K_2_MnPc is reached. This nicely corroborates that the lowest-lying unoccupied MnPc orbital that is filled by electrons, has predominantly Mn 3d character [22,72,84], whereas for the other phthalocyanines a ligand π^*^ state is filled right from the beginning.

**Figure 4:**
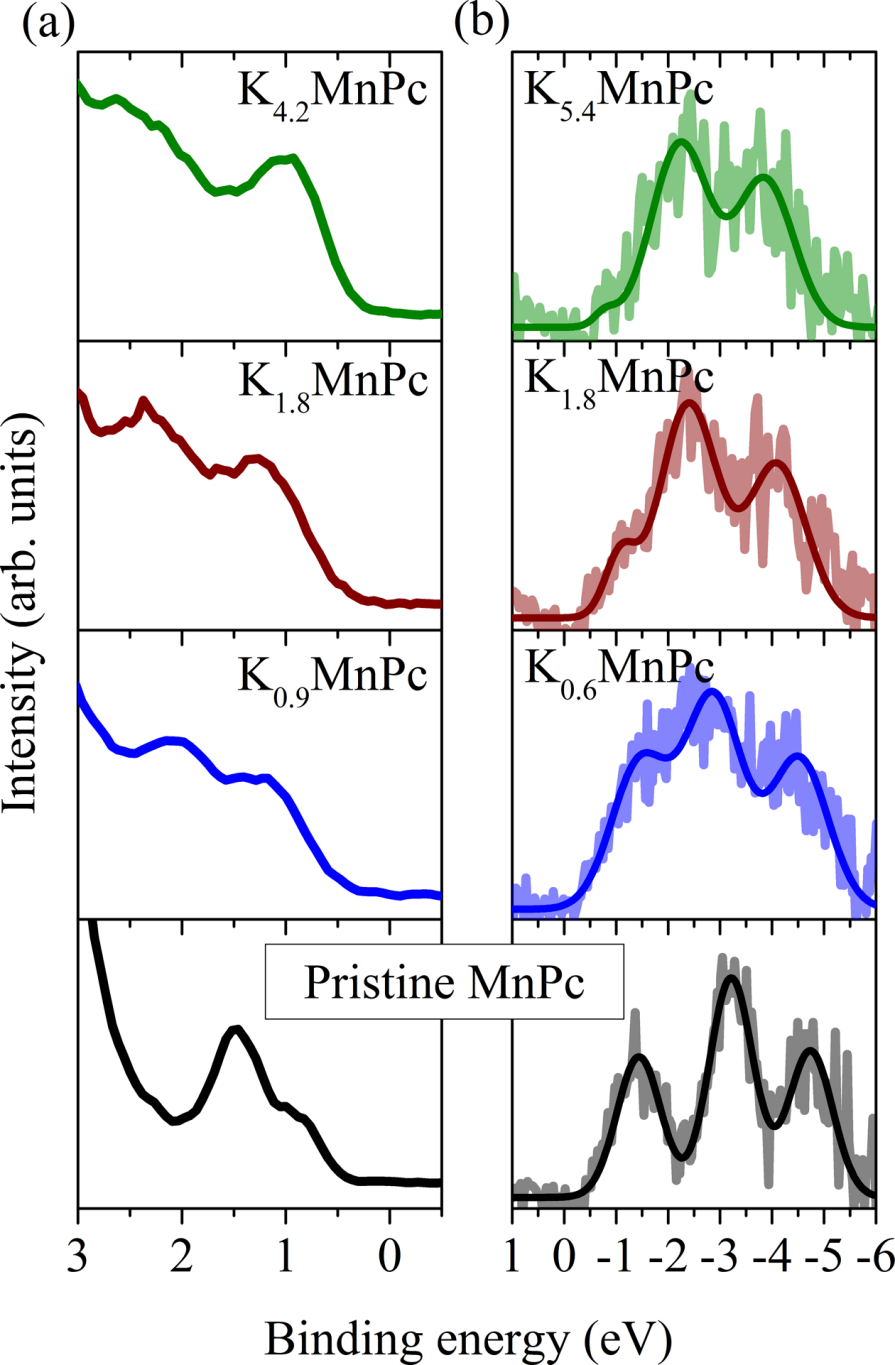
Panel (a): valence band photoelectron spectroscopy results for undoped and three potassium-doped MnPc films, which represent the occupied density of states. The doping levels were determined using PES from C 1s and K 2p core levels [49]. Panel (b) depicts data from inverse photoelectron spectroscopy, i.e., the unoccupied density of states.

The results shown above already indicate that all of the potassium-doped MnPc phases are characterized by an energy gap. This observation is in full agreement with the results of photoelectron spectroscopy studies and inverse photoelectron studies of K-doped MnPc [49] as discussed in the following. The samples for these investigations again were prepared by potassium addition to MnPc thin films in ultra-high vacuum. The doping level of the films was determined by analyzing the relative intensities of the core level photoemission from the C 1s and the K 2p core levels [49]. In Figure 4 we summarize the results of PES and IPES data that could be obtained for compositions close to the K*_x_*MnPc phases that were discussed above.

We start the discussion with the spectra for undoped MnPc. The PES data in Figure 4a show the well-known two maxima close to the chemical potential (0 eV binding energy) [18,22]. They arise from emission from the highest occupied molecular orbital (HOMO) at about 1.5 eV binding energy, and from the so-called SOMO (singly occupied molecular orbital) at about 0.7 eV. Going to K_0.9_MnPc, there is an energy shift to higher binding energies, which is due to a shift of the Fermi level towards the unoccupied levels. Furthermore, the feature at lowest binding energy grows in intensity, which reflects the filling of the SOMO with one more electron. Adding more potassium (K_1.8_MnPc) results in a further shift of the maxima to somewhat higher energies, while the spectral onset moves slightly downward. This downshift is related to the filling of the former LUMO with electrons, i.e., a new occupied state appears in the photoemission data. Further doping to K_4.2_MnPc causes an intensity increase at low binding energy, which results from further filling of the former LUMO.

Figure 4b presents information on the unoccupied electronic states as measured using IPES [49]. For undoped MnPc the spectrum represents the first three unoccupied levels, the LUMO (lowest unoccupied molecular orbital), the LUMO+1, and the LUMO+2. We note that the IPES data do not reveal the unoccupied part of the SOMO (see above), which is attributed to the limited energy resolution of the data. The formation of K*_x_*MnPc phases results in an intensity decrease of the feature representing the LUMO due to electron addition into this orbital, in agreement to the discussion above. Importantly, independent of the potassium concentration both PES and IPES data, in agreement with the EELS data above demonstrate a clear energy gap, i.e., none of the K*_x_*MnPc phases is metallic. This, in general, resembles the situation in many molecular crystals doped with alkali metals, where it was observed that the doping did not result in a metallic ground state although metallicity would be expected on the basis of band-structure calculations since half-filled bands are present. Molecular crystals usually have energy bands with small band widths, which is a direct consequence of the rather small interaction between the molecules in the material. Furthermore, the bandwidth often is similar to the Coulomb repulsion of two charge carriers on one molecule. Thus, molecular crystals also are correlated materials, where the electronic correlations often are strong enough to induce an insulating Mott–Hubbard ground state [85–94].

### MnPc cations in an organic salt: MnPc/F_4_TCNQ

In the following we present information on the electronic properties of a purely organic salt in which MnPc is oxidized. In order to obtain such a compound we have prepared mixed films consisting of MnPc and the particularly strong electron acceptor F_4_TCNQ. It is well known that high-quality organic charge transfer crystals with F_4_TCNQ as electron acceptor can be grown [95–103]. Phthalocyanine-based compounds, however, have not been reported yet. We prepared the mixed films by evaporating F_4_TCNQ on top of a MnPc film and taking advantage of the diffusion of F_4_TCNQ into the MnPc film, or by simultaneously evaporating both materials [104]. Subsequently, we have applied an in situ distillation procedure, which was already applied in previous experiments to achieve stoichiometric molecular salts [105–108]. After the initial preparation step, the films were heated up to 340 K for about half an hour, where an evaporation of surplus F_4_TCNQ could be seen even with bare eye [104]. Also, this procedure resulted in the formation of MnPc/F_4_TCNQ films with a well-defined composition and with well-defined spectral response, which signalled the homogeneity of the films. The composition was analyzed using the relative intensities of photoemission core-level features [104], and in all cases we obtained a stoichiometric ratio of 1:1.

**Figure 5:**
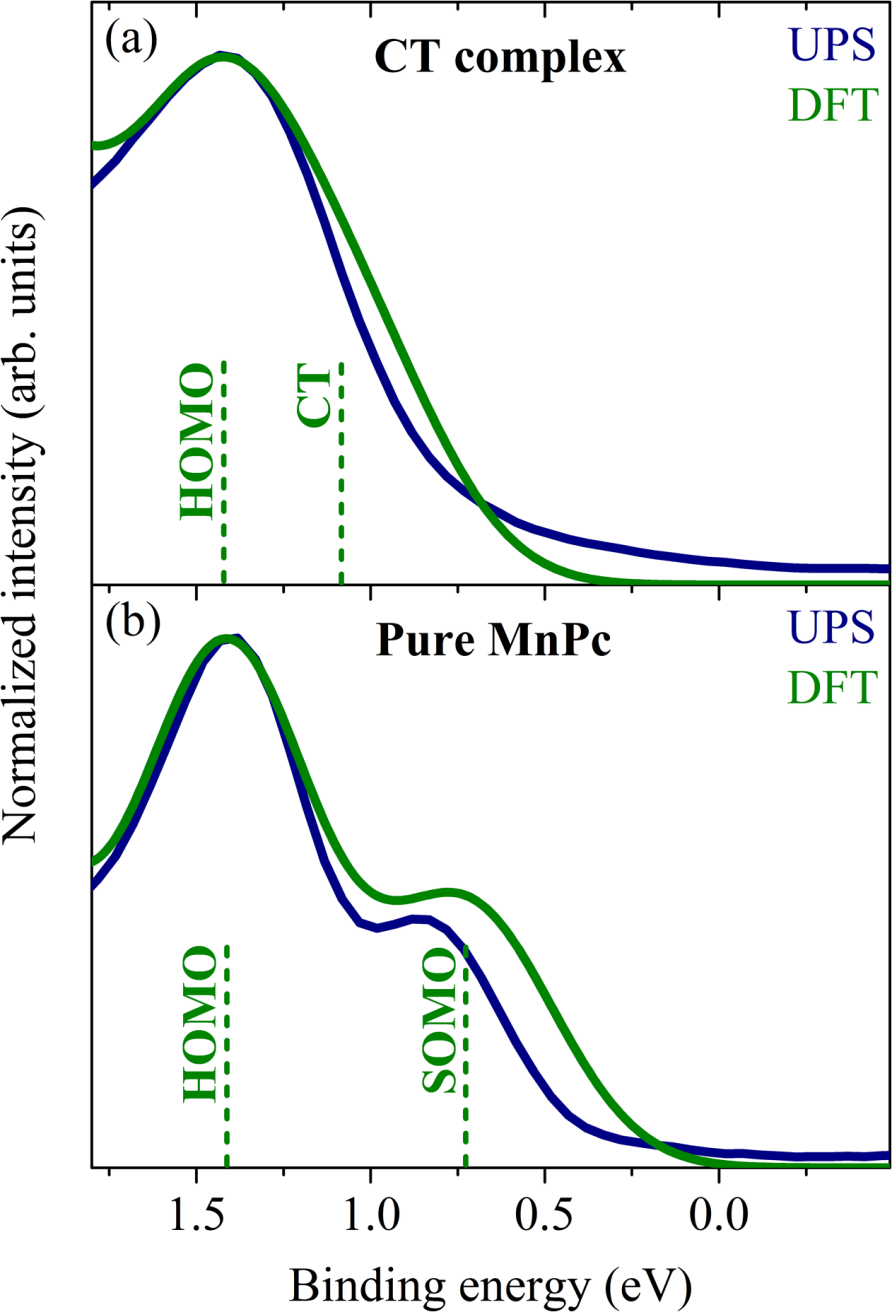
Comparison of valence band photoelectron spectroscopy (UPS) data to those from density functional based calculations (DFT) for pure MnPc (lower panel) and the charge-transfer compound MnPc/F_4_TCNQ (upper panel). The vertical bars denote the energy position of the molecular orbitals as determined by the calculations. For pure MnPc the two-peak structure arises from the singly occupied molecular orbital (SOMO) and the highest occupied molecular orbital (HOMO). The formation of the charge-transfer (CT) compound leads to a disappearance of the SOMO. Instead, a CT hybrid state shows up, closer in energy to the HOMO (see also [104]).

We have analyzed the electronic properties of the new organic charge-transfer compound MnPc/F_4_TCNQ using photoemission and electron energy-loss spectroscopy as well as density functional theory based calculations. In Figure 5 we show the PES results in the valence band region.

**Figure 6:**
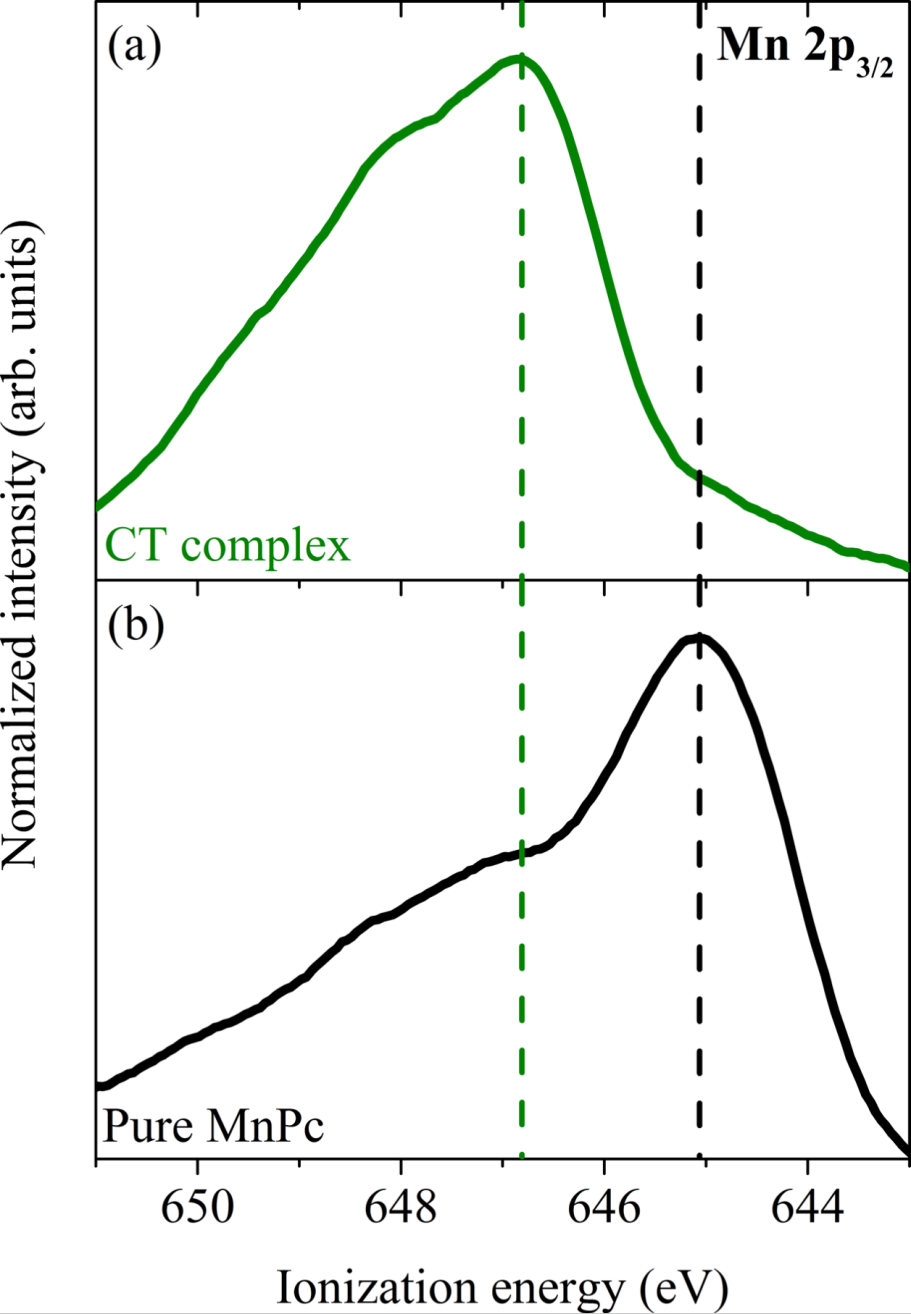
Core-level photoelectron spectroscopy data in the energy region of the Mn 2p_3_*_/_*_2_ core level (adapted from [104]). Note that the energy is referenced to the vacuum level in this case, since two different compounds with different Fermi-level positions are compared. Panel (a) shows the core-level emission of the MnPc/F_4_TCNQ charge-transfer compound, while panel (b) shows that of pure MnPc. Clearly, the formation of MnPc/F_4_TCNQ results in an energy shift of about 1.8 eV to higher ionization energy.

These data clearly illustrate a substantial variation going from pure MnPc to the charge-transfer compound. For pure MnPc our data demonstrate the well-known two peak structure at lowest binding energy (see also the previous chapter). The formation of the MnPc/F_4_TCNQ compound is accompanied by the disappearance of the feature at lowest binding energy (here called SOMO = singly occupied molecular orbital). This indicates that electrons are removed from MnPc, i.e., the phthalocyanine molecule is oxidized. Since the leading orbital in pure MnPc (SOMO) is a hybrid state of Mn 3d and ligand π orbitals with a large Mn 3d contribution [22,84,109], this oxidation is also clearly seen in core-level photoemission from MnPc 2p core level states as depicted in Figure 6.

This figure presents the comparison of the Mn 2p_3_*_/_*_2_ core-level emission spectrum from a pure MnPc film and from the charge-transfer compound MnPc/F_4_TCNQ. The overall spectral shape is controlled by an underlying multiplet structure [110–111] and is not discussed here further. For MnPc/F_4_TCNQ the Mn 2p core level is significantly shifted to a higher ionization energy by about 1.8 eV, which results from the oxidation of the central Mn atom in MnPc. Shifts of the Mn 2p photoemission core-level features to higher energies were also observed going from MnO to, e.g., Mn_3_O_4_ [112], where also the number of Mn 3d electrons is reduced. Thus, the core-level data corroborate our conclusion above.

**Figure 7:**
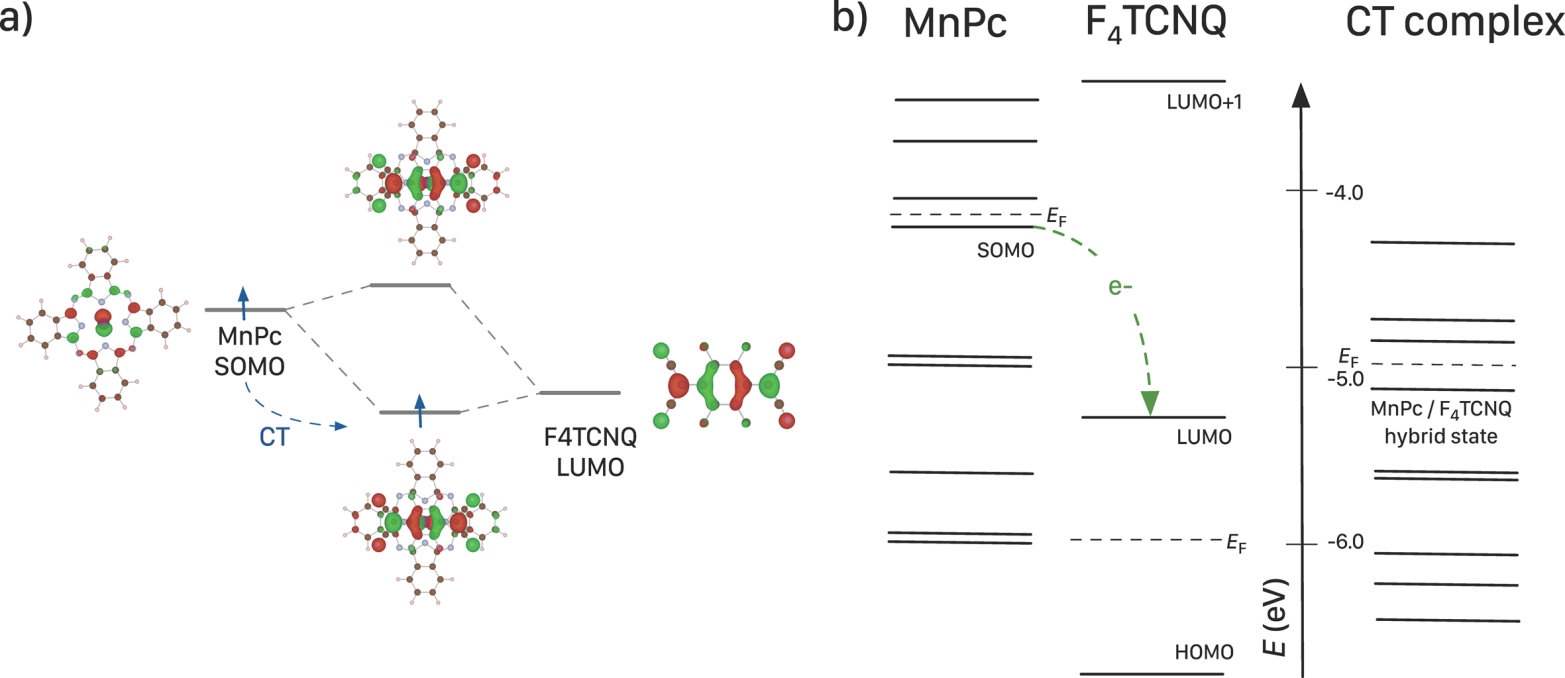
Results of the DFT calculations for the MnPc/F_4_TCNQ dimer model systems: a) The SOMO of MnPc and the LUMO of F_4_TCNQ hybridize and charge is transferred into the newly formed bonding hybrid state. b) Comparison of the eigenvalues of the Kohn–Sham orbitals as obtained from the calculations for a single MnPc molecule, a single F_4_TCNQ molecule and the dimer model compound (for more information see [104]).

The charge-transfer reaction between MnPc and F_4_TCNQ was also studied on the basis of dimer calculations. These calculations indicate the formation of a hybrid state between the highest occupied orbital of MnPc (here called SOMO for singly occupied molecular orbital) and the LUMO of F_4_TCNQ, see Figure 7. The energy position of this hybrid state is lower than that of the SOMO of MnPc. In addition, the calculations demonstrate a charge transfer between the molecules of about 0.6 electrons per dimer. The experimental results indicate an even larger charge transfer, which most likely is related to the localization error inherent to all DFT semi-local exchange correlation functionals [113]. Nevertheless, the calculations provide a reasonable understanding of the occupied electronic states of MnPc/F_4_TCNQ as demonstrated in Figure 5. Both the disappearance of the spectral feature at lowest binding energy of MnPc as well as the broadening of the structures is well reproduced. The calculations also indicate that the hybrid state is too close to the MnPc HOMO to be resolved spectroscopically.

**Figure 8:**
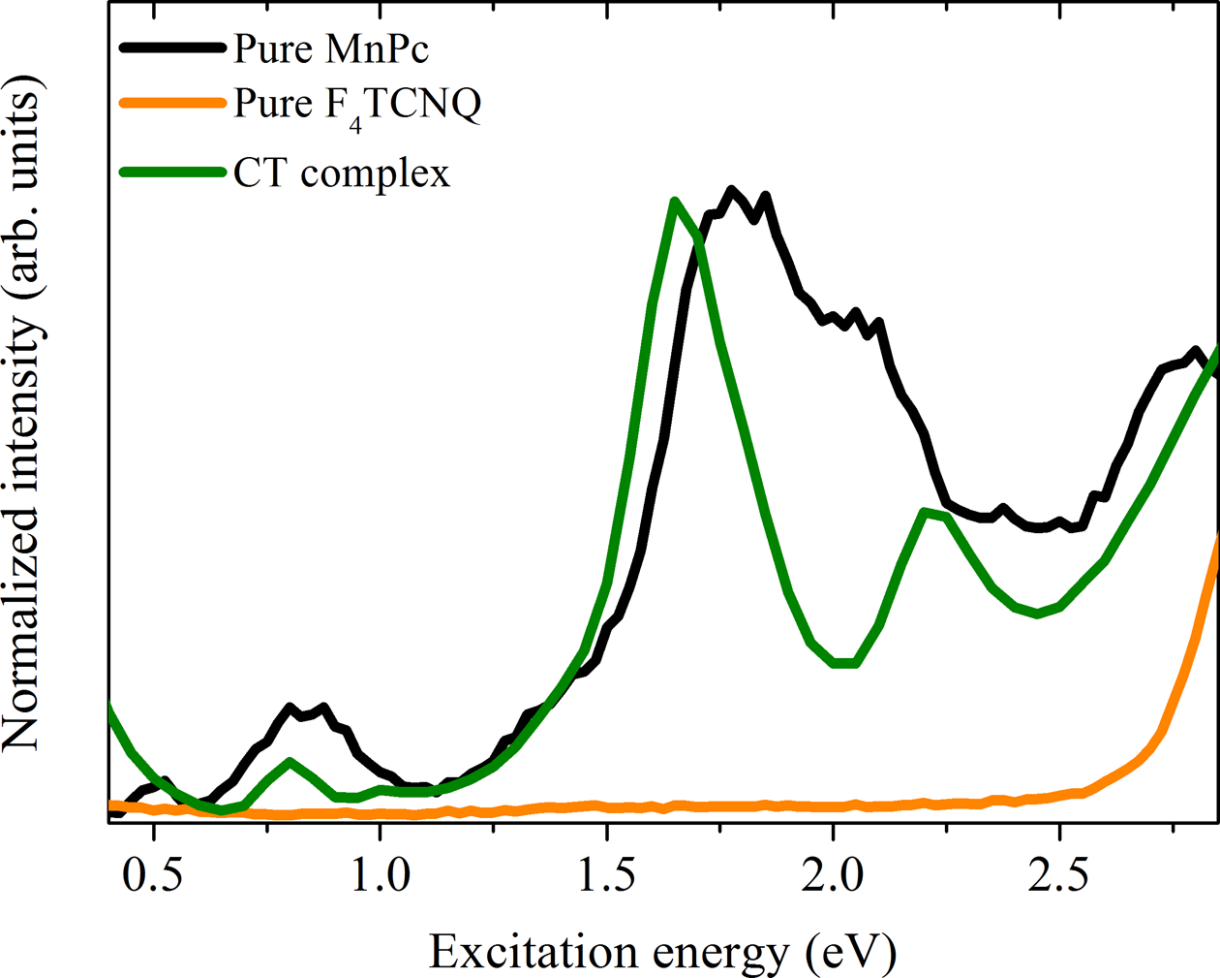
Comparison of the electronic excitation spectra of MnPc, F_4_TCNQ and the charge-transfer compound MnPc/F_4_TCNQ (adapted from [104]).

The formation of the charge-transfer complex MnPc/F_4_TCNQ also results in corresponding changes of the electronic excitation spectra in comparison to those from pure MnPc or F_4_TCNQ. This is illustrated in Figure 8, where the corresponding data from EELS are depicted. The measured spectrum of pure F_4_TCNQ is characterized by a rather large energy gap and an excitation onset at about 2.7 eV that is followed by a broad structure around 3.3 eV [114–115]. The rather complex excitation spectrum of MnPc was already discussed above. The formation of the charge-transfer compound MnPc/F_4_TCNQ gives rise to clearly different electronic excitations. The lowest excitation feature as seen for pure MnPc at about 0.5 eV cannot be seen any more. This can be associated to the removal of an electron from the leading orbital of MnPc as discussed above. The excitation spectrum of MnPc/F_4_TCNQ consists of rather sharp excitation maxima at around 0.8 eV, 1.65 eV and 2.2 eV. A detailed quantitative description of these excitations has not been achieved yet [104]. In general, our data show that the MnPc/F_4_TCNQ compound has an energy gap of about 0.6 eV represented by the spectral onset in the excitation data. In particular, the lowest energy (gap) excitation is ascribed to the excitation within the two-level system, which originates from the charge-transfer reaction and the related hybrid-state formation as discussed above.

**Figure 9:**
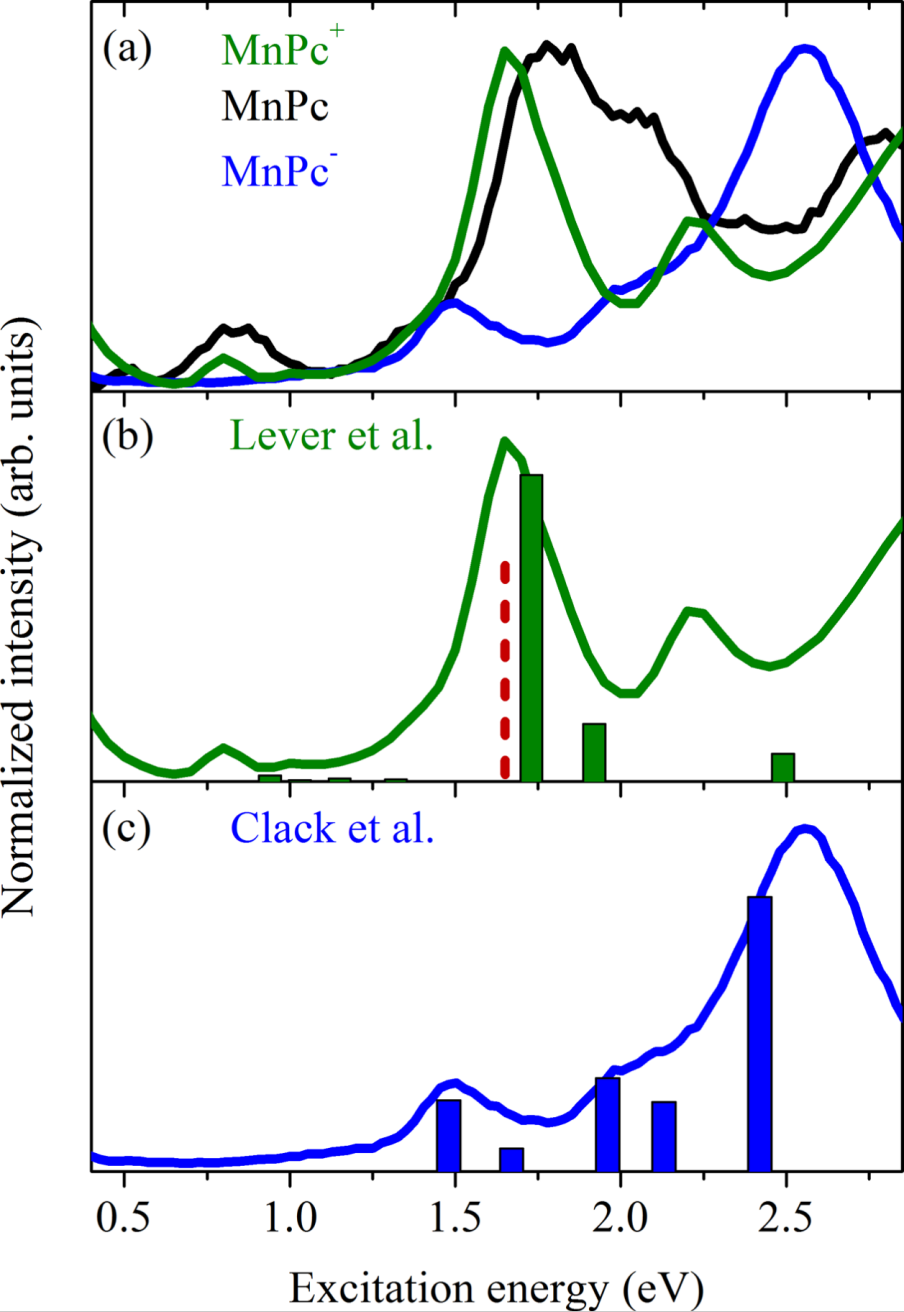
(a) Comparison of the electronic excitation spectra of MnPc, K_1_MnPc and MnPc/F_4_TCNQ as measured using EELS. In the lower two panels, we compare these data to the optical absorption energies (denoted by vertical bars) as observed for (b) MnPc^+^ [117] and (c) MnPc^−^ [118] in solution. In panel (b) we additionally show the optical absorption energy of F_4_TCNQ^−^ radicals in solution (dashed line) [116].

Thus, the oxidation of MnPc molecules upon the formation of the new charge transfer salt MnPc/F_4_TCNQ is clearly seen in our spectroscopic data and supporting calculations. Together with the results on potassium-doped MnPc as presented in the previous chapter, this nicely demonstrates the variability of MnPc in charge-transfer compounds, where it can be either reduced or oxidized. We conclude the discussion of the MnPc-based charge-transfer compounds with a comparison of the electronic excitation spectra of MnPc, oxidized MnPc^+^ and reduced MnPc^−^ as measured for the MnPc/F_4_TCNQ and K_1_MnPc compounds. We argue that for both compounds the low-energy excitations predominantly stem from MnPc-derived orbitals. In the case of K_1_MnPc this is quite clear since potassium ions do not contribute in the relevant energy region. For MnPc/F_4_TCNQ the situation is more complex. Based on a purely ionic picture, also excitations from the negatively charged F_4_TCNQ should show up. In solution, an excitation for F_4_TCNQ^−^ radicals at about 1.65 eV was reported [116]. Keeping this in mind, we present a comparison of our electronic excitation data obtained using EELS for MnPc, K_1_MnPc (MnPc^−^), and MnPc/F_4_TCNQ (MnPc^+^) in Figure 9. In addition, we also included the energies of optical absorption studies for oxidized and reduced MnPc in solution [117–118]. Taking into account a broadening upon transition from single molecules in solution to the solid state as well as energy shifts due to different polarization screening, there is very good agreement between our data and those in solution from the literature. This nicely corroborates the interpretation of our results in terms of MnPc salts and the related electronic properties.

### MnPc/F_6_TCNNQ: charge transfer at an interface

The organic heterojunction MnPc/F_6_TCNNQ represents an example, where MnPc is involved in a charge transfer across an interface. In consideration of the results of the previous chapter, it is reasonable to expect charge transfer between MnPc and F_6_TCNNQ, since F_6_TCNNQ is an even stronger electron acceptor compared to F_4_TCNQ. Moreover, it is larger and heavier, which prevents it from diffusion in or into organic films. Thus, it will form well-defined interfaces, i.e., also the charge-transfer reaction is confined to the interface region.

In general, charge transfer from insulators or semiconductors on one side of the interface to those on the other side can have dramatic effects and result in interfacial electronic properties that differ substantially from those of the individual materials [32,119–124]. For instance, particular interfaces between two initially semiconducting organic materials were shown to become even metallic [32,120].

**Figure 10:**
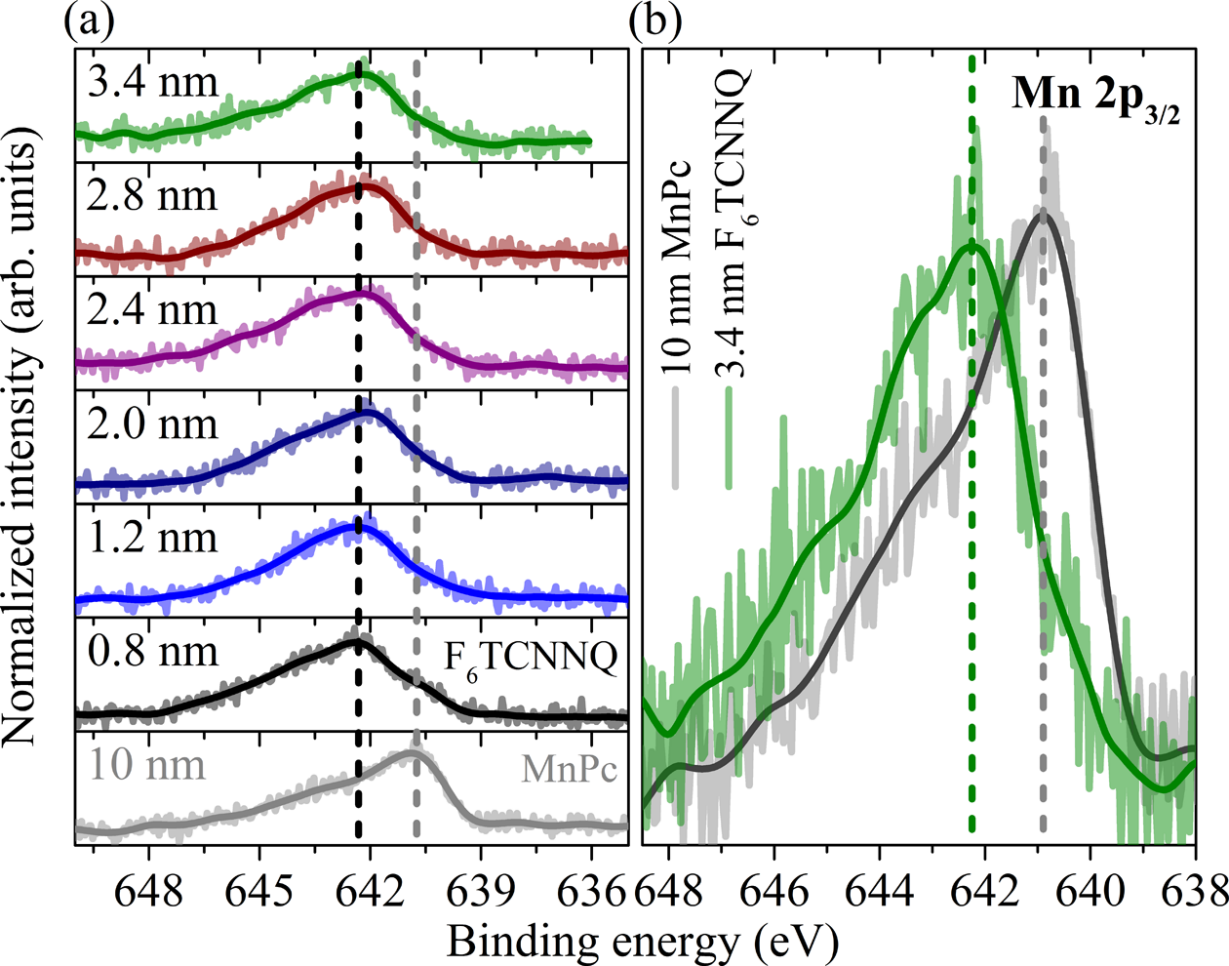
Core-level photoelectron spectroscopy data in the energy region of the Mn 2p_3_*_/_*_2_ core level for the MnPc/F_6_TCNNQ interface. Panel (a) shows the data as a function of F_6_TCNNQ thickness on top of MnPc (as indicated), while panel (b) compares the Mn 2p_3_*_/_*_2_ features for pure MnPc and with a 3.4 nm top layer of F_6_TCNNQ. There is a clear energy shift of 1.5 eV to higher binding energy.

First photoemission studies of the MnPc/F_6_TCNNQ interface indeed suggest that there is substantial charge transfer, which might also lead to interesting physics at these interfaces. In Figure 10 we show the results of the measurements of the Mn 2p_3_*_/_*_2_ core-level emission during the formation of the MnPc/F_6_TCNNQ interface. Here, F_6_TCNNQ was deposited stepwise onto a 10 nm thick MnPc film. Due to the rather small electron escape depth (a few angstroms only [39]) the Mn 2p_3_*_/_*_2_ core-level data increasingly stem from regions very close to the interface with increasing F_6_TCNNQ top layer thickness. The data in Figure 10 demonstrate a clear change in line shape and energy position of the the Mn 2p_3_*_/_*_2_ core-level feature, which is analogous to the changes seen above for MnPc/F_4_TCNQ. Thus, these data evidence that there is charge transfer at the MnPc/F_6_TCNNQ interface and that the Mn central atom in MnPc again is oxidized.

**Figure 11:**
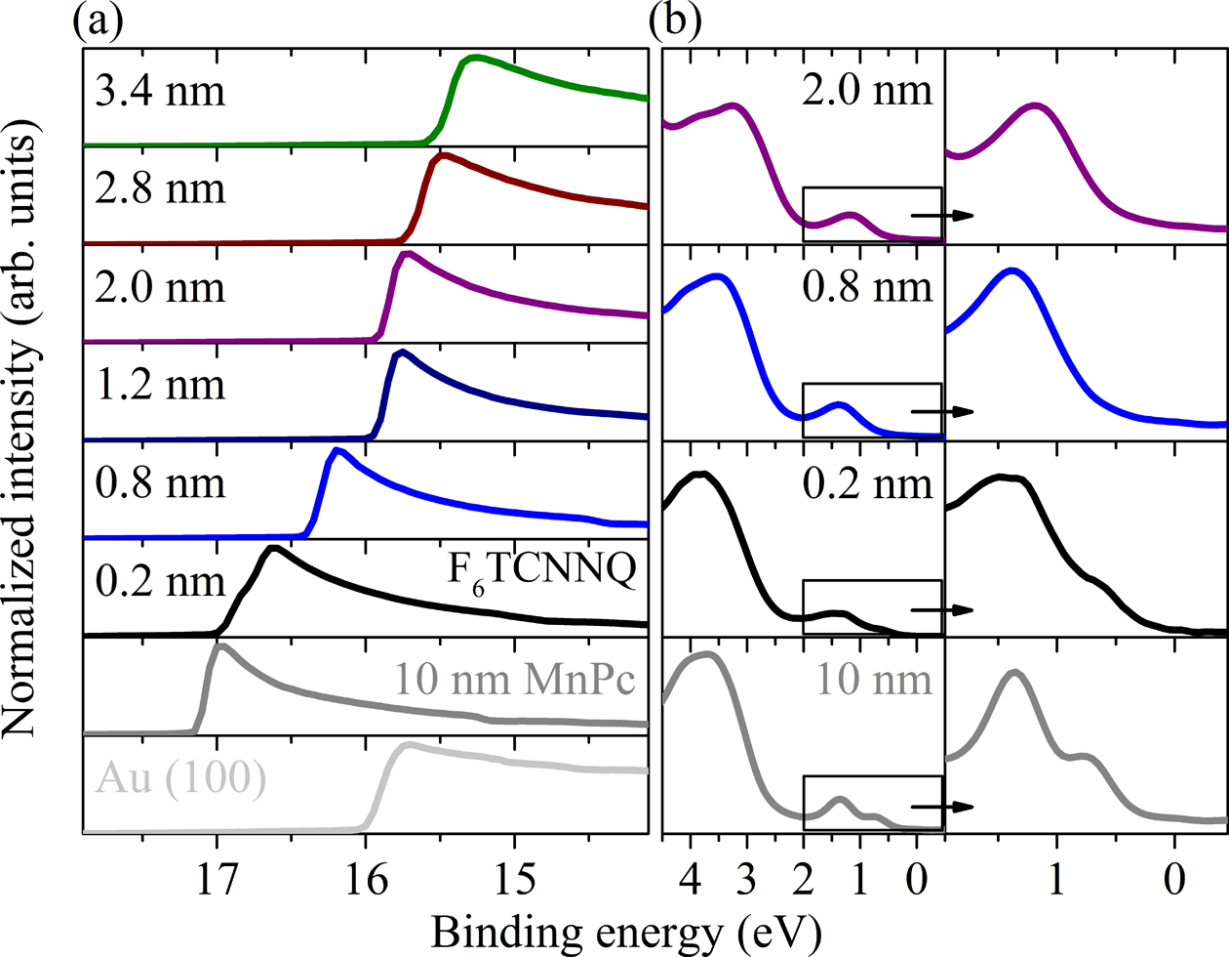
Valence-band photoemission data from the MnPc/F_6_TCNNQ interface as a function of the F_6_TCNNQ top-layer thickness. Panel (a) shows the evolution of the secondary electron cutoff, which represents the changes of the work function. Panel (b) focusses on the energy region close to the Fermi level.

Furthermore, also the valence band data clearly indicate this charge transfer. Figure 11 illustrates the evolution of the high binding energy cutoff (a) and the energy region close to the Fermi energy (b). The data in panel (a) show the evolution of the work function of the layer system as a function of increasing F_6_TCNNQ layer thickness. With the exception of the thinnest F_6_TCNNQ layer on top of MnPc, the well-pronounced and sharp cutoff spectra affirm the formation of well-defined organic layers. In the case of 0.2 nm F_6_TCNNQ, a step is visible in the region of the secondary cutoff, which most likely is due to a coverage of less than a monolayer F_6_TCNNQ on MnPc, which results in surface/interface regions with and without the charge-transfer reaction. Figure 11b depicts the data close to the Fermi level for a selection of F_6_TCNNQ overlayer thicknesses. Again, for pure MnPc the spectrum is characterized by a two peak feature as described above. The features at lowest binding energy (about 0.7 eV) vanishes when F_6_TCNNQ is added, which signals the oxidation of MnPc in analogy to the previous section. The second feature, initially at about 1.4 eV, first broadens, then becomes somewhat sharper again and shifts to 1.2 eV. We attribute this feature to the emission from the now filled, formerly lowest unoccupied molecular orbital of F_6_TCNNQ, which is occupied at the interface as a result of the charge transfer. Consequently, our data provide evidence for a considerable charge transfer at the MnPc/F_6_TCNNQ interface, and further investigations are necessary to provide more insight into the physics as, e.g., whether there is electrical conduction along the interface.

### A charge and spin transfer interface: MnPc/F_16_CoPc

A further example in which a charge transfer across an interface results in new physical properties at this interface is provided by bringing together MnPc and F_16_CoPc. Highly ordered interfaces of this kind were realized by depositing one of the two phthalocyanines on a gold(100) single crystal, which resulted in well-oriented thin films [125–127]. Subsequently, the partner phthalocyanine was deposited on top, which finally gave rise to well-ordered heterojunctions as revealed by polarization dependent X-ray absorption spectroscopy (XAS) studies [127].

**Figure 12:**
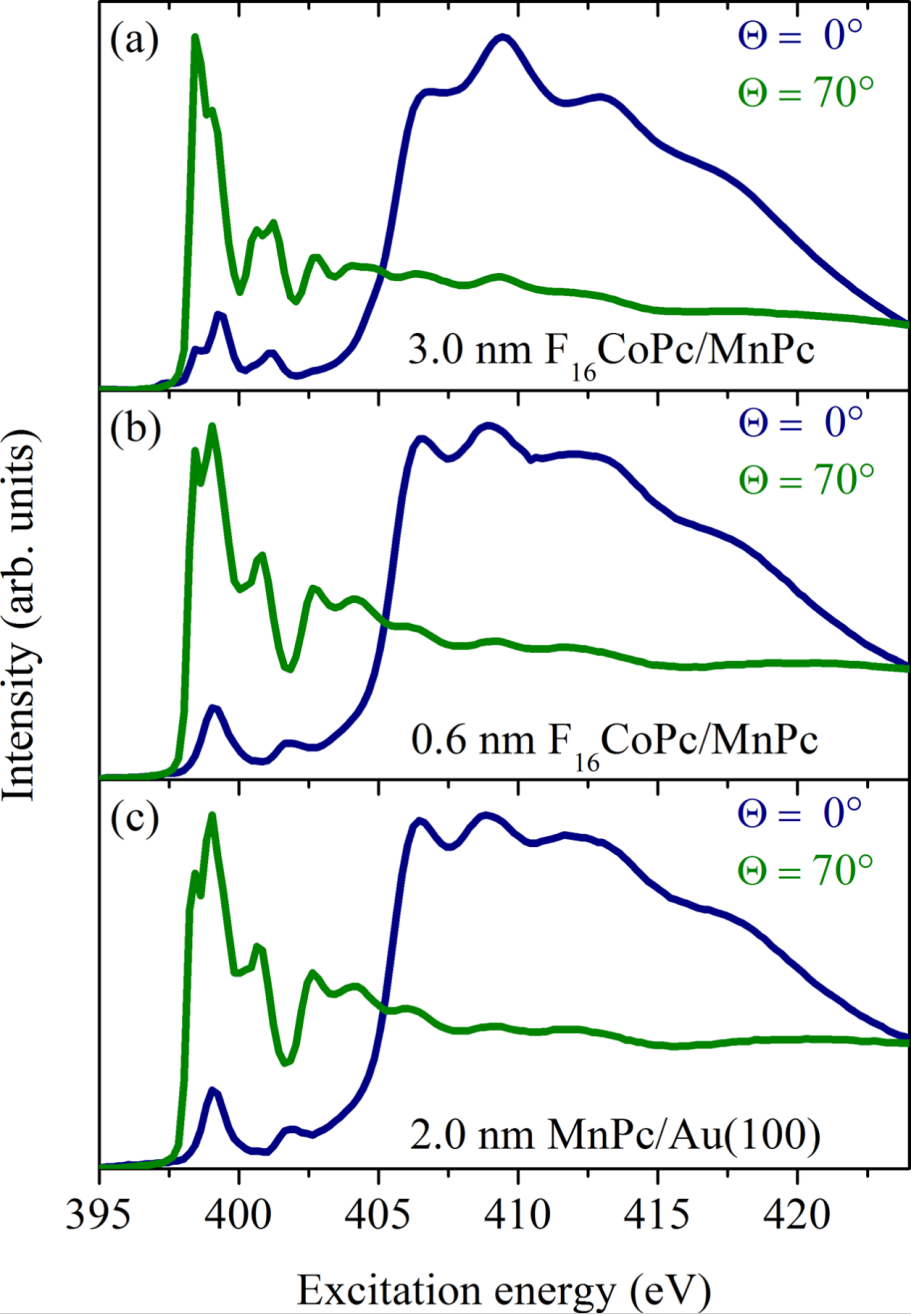
N 1s excitation spectra as obtained using X-ray absorption spectroscopy (adapted from [127]). Corresponding data for (c) a 2.0 nm thick MnPc film on Au(100), (b) an additional 0.6 nm thick F_16_CoPc overlayer on MnPc, and (a) a relatively thick F_16_CoPc overlayer (3 nm) are depicted. The spectra were recorded with two different angles of beam incidence. Θ denotes the angle between the surface normal and the direction of the incident beam. The incoming radiation is linearly polarized.

In Figure 12 we present corresponding N 1s absorption spectra for pure MnPc and two different film thicknesses of an F_16_CoPc overlayer, deposited on a gold single crystal. Different light polarizations with respect to the film surface normal were achieved by variation of the angle between the incident light and the surface normal (see angles in Figure 12). For phthalocyanines it is well known that 1s→σ^*^ and 1s→π^*^ excitations take place for a light polarization vector perpendicular and parallel to the molecular planes, respectively. Moreover, previous studies [128–129] have demonstrated that the relatively sharp N 1s excitation features around 398 eV are due to transitions from the N 1s core level into the unoccupied π^*^ orbitals with N 2p orbital contributions, which are oriented perpendicular to the molecular plane. The higher energy structures above 405 eV are related to N 1s→σ^*^ transitions.

**Figure 13:**
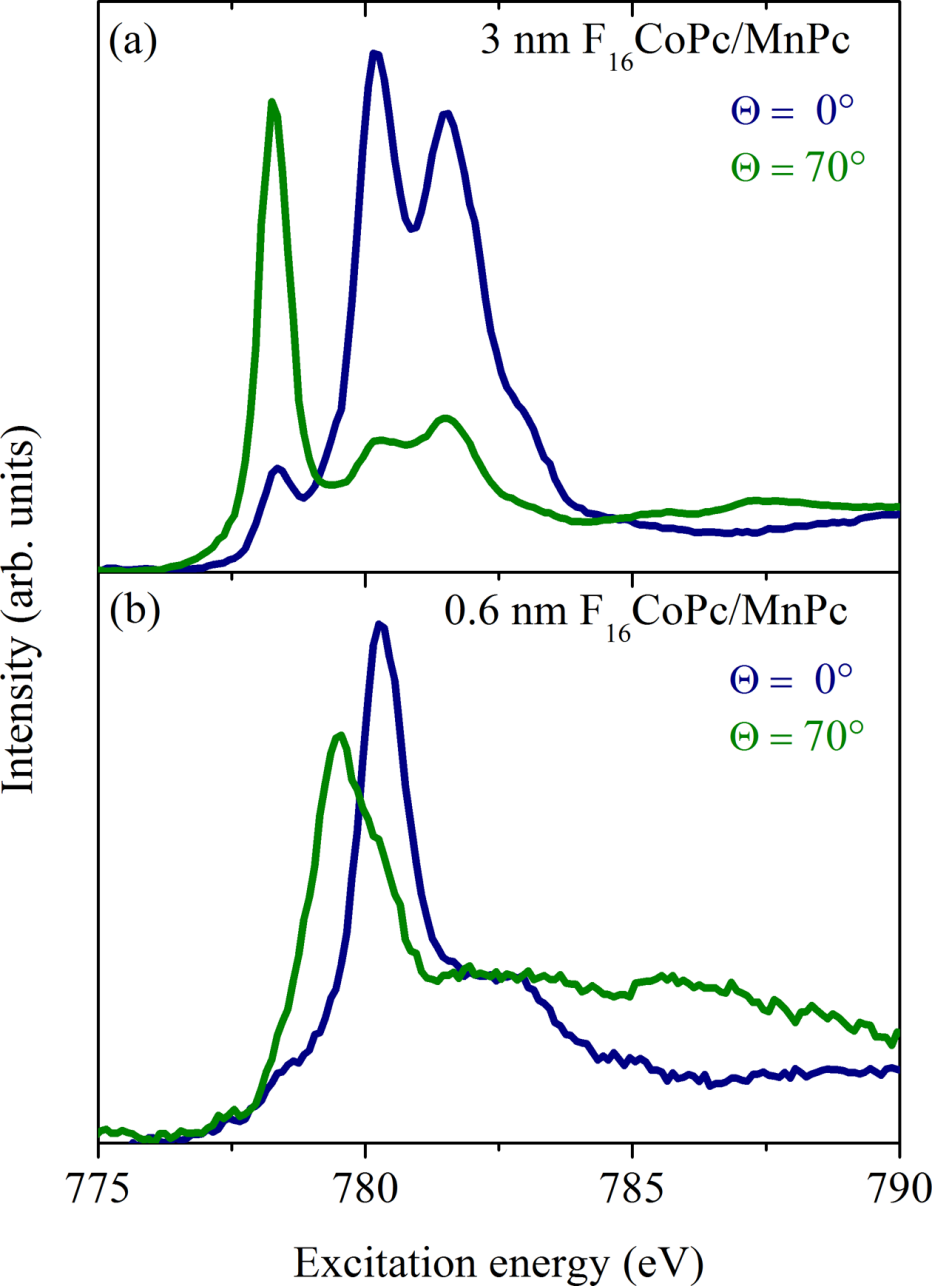
Polarization dependent X-ray absorption data at the Co L_3_ edge for a (a) 3 nm and (b) 0.6 nm F_16_CoPc overlayer on MnPc (adapted from [127]). Again, the incident beam direction is given by the angle Θ (see Figure 12 above).

The data shown in Figure 12 reveal a very strong polarization dependence for the absorption edges of pure MnPc. The observed intensity variations show that the respective phthalocyanine molecules are arranged parallel to the substrate surface with a very high degree of orientation. The mean deviation from exactly parallel lying molecules is only about 5% [127]. This very high degree of order is also kept across the MnPc/F_16_CoPc interface, as can be seen from Figure 12. In other words, the two phthalocyanines form an interface where they lie face to face. This high degree of order also allowed us to study the anisotropy of the excitations into Co-derived 3d levels in F_16_CoPc close and far from the interface to MnPc. The corresponding data at the Co 2p absorption edges again show a very clear anisotropy when taken with different polarization directions of the incident synchrotron radiation, as depicted in Figure 13.

The data for a 3 nm thick F_16_CoPc film on top of MnPc are very similar to the corresponding absorption spectra of pure CoPc on gold [130]. This indicates that fluorination of CoPc has little impact on the electronic 3d states of the central Co atom. The lowest lying absorption feature, which is maximal for a light polarization perpendicular to the F_16_CoPc molecules, can be assigned to transitions from the Co 2p into unoccupied 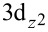 states. The higher lying features stem from a multiplet structure related to the excitations into the Co 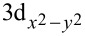 orbital [130].

For thin F_16_CoPc films deposited onto MnPc we observe considerably different spectra. Features that are characteristic for pure F_16_CoPc disappear while new structures show up around 780 eV, which are still anisotropic. This provides clear evidence that there is a reaction between F_16_CoPc and MnPc at the corresponding interface, which affects the cobalt states of F_16_CoPc. This conclusion is supported by equivalent investigations of CoPc monolayers on gold and silver surfaces, where similar changes in the absorption spectra were observed and where a charge transfer between the cobalt 3d states and the underlying metallic substrate occurs [131–132]. Moreover, also the Mn L_2,3_ absorption edge is subject to substantial changes for MnPc molecules in contact to F_16_CoPc in comparison to pure MnPc [127]. This provides clear evidence that the charge transfer at the MnPc/F_16_CoPc interface again results in charge removal from Mn 3d orbitals in MnPc.

**Figure 14:**
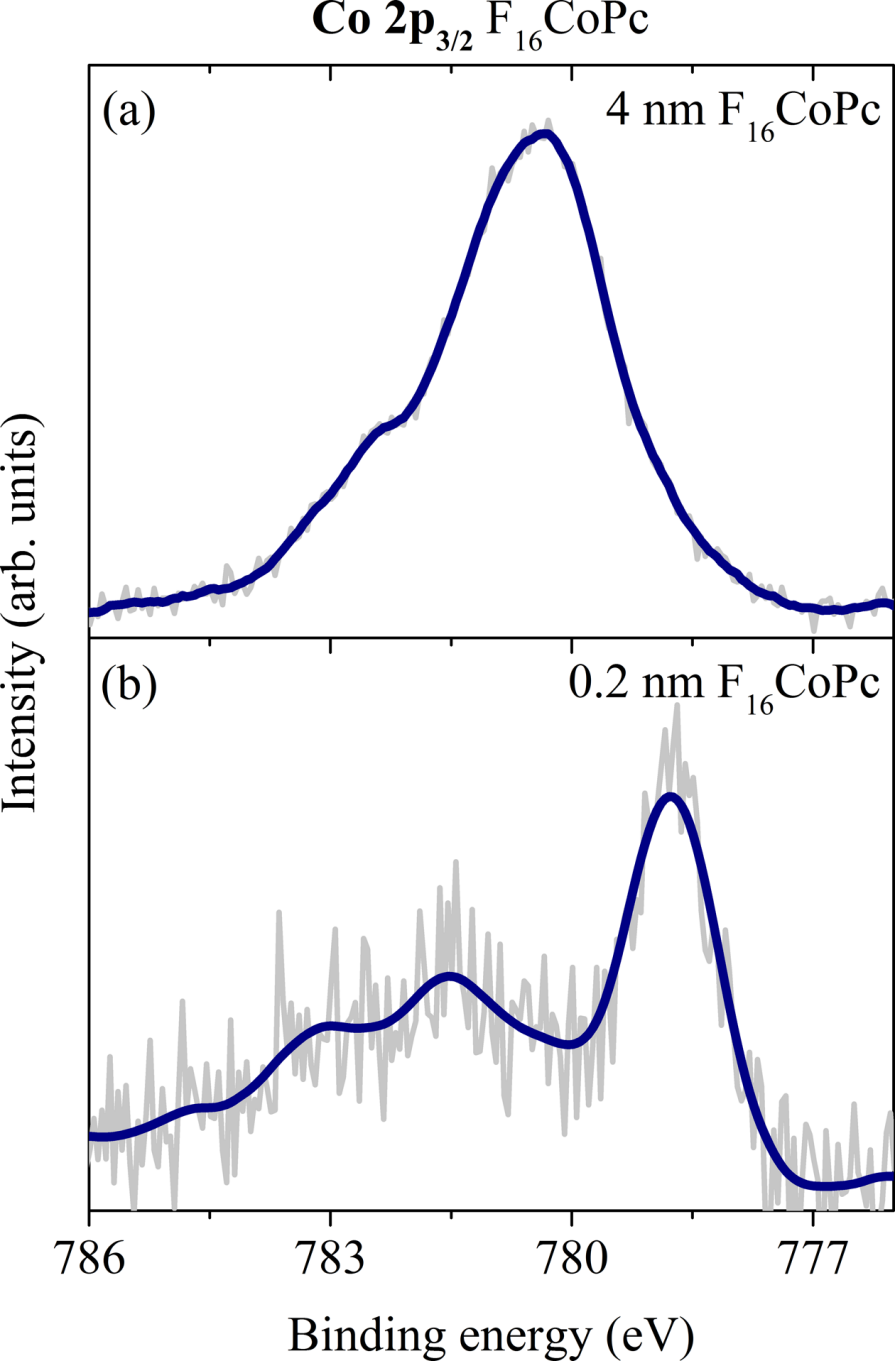
Co 2p_3_*_/_*_2_ core-level photoemission spectra of a (a) thick and (b) thin F_16_CoPc layer on top of MnPc. The appearance of a structure of low binding energy for the thin F_16_CoPc layer clearly indicates a reduction of the Co center in this molecule (adapted from [31]).

A charge-transfer reaction at the MnPc/F_16_CoPc interface is also seen in photoelectron spectroscopy studies [31]. Looking at the Co 2p_3_*_/_*_2_ core-level data of this interface, there is a significant change in line shape and binding energy as a function of the F_16_CoPc layer thickness. These data are shown in Figure 14.

**Figure 15:**
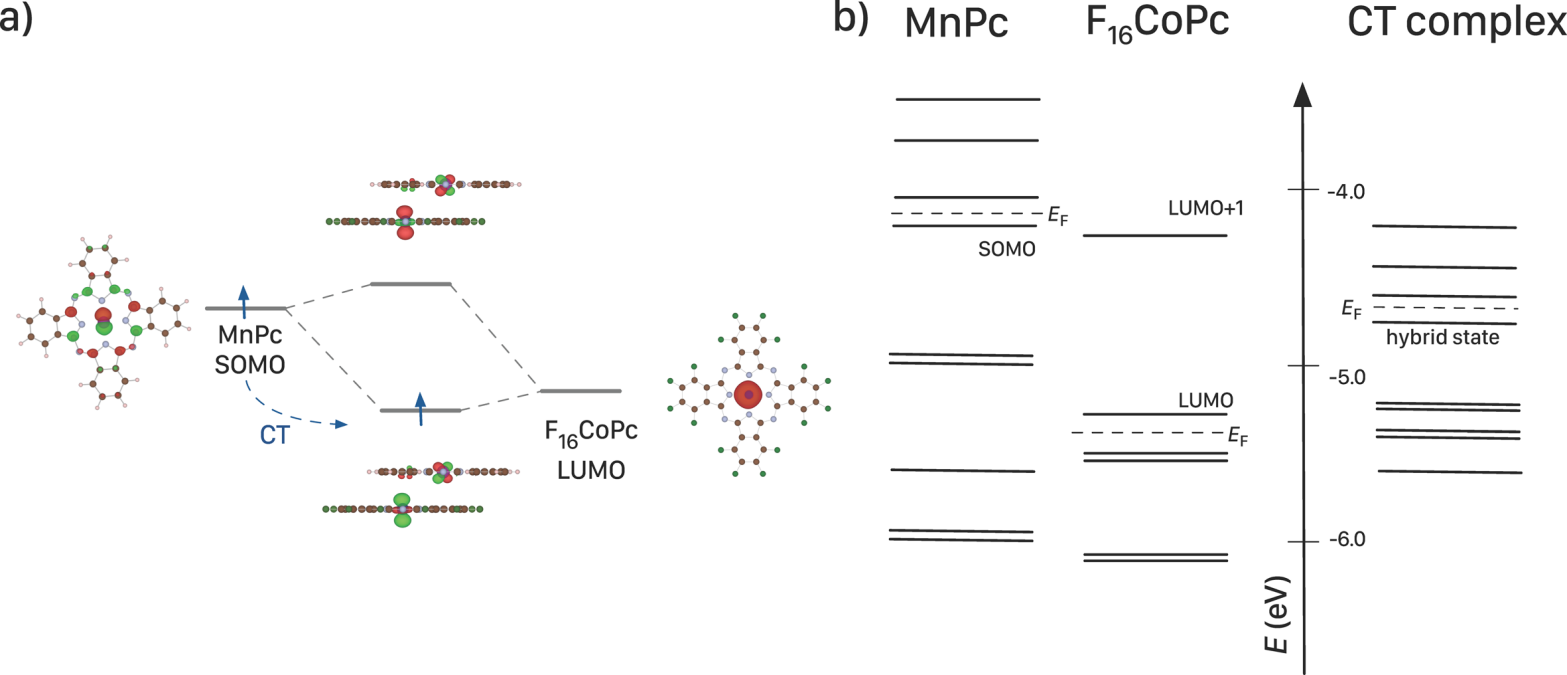
Results of the DFT calculations for the MnPc/F_16_CoPc model systems: a) The hybrid state is formed by the Mn 3d*_xz_* and the Co 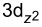 states. b) Comparison of the eigenvalues of the Kohn–Sham orbitals as obtained from the calculations for a single MnPc molecule, a single F_16_CoPc molecule and the dimer model (complex).

The data for the thin F_16_CoPc layer are rather similar to what has been reported so far for cobalt porphyrines and phthalocyanines deposited on various metals. In these cases, a relatively strong interaction of the Co center of the molecules and the metal surface takes place [133–137]. The result of this interaction usually is rationalized in terms of a reduction of the metal center to Co(I). Consequently, there is clear evidence that at the MnPc/F_16_CoPc interface the Co center of F_16_CoPc is also reduced as a result of a charge transfer across this interface. Also, a corresponding shift of the Mn 2p core-level feature to a higher binding energy is observed [31] (cf. Figure 6). The charge transfer arises from the formation of hybrid states between the transition-metal centers of the two phthalocyanines, with a concomitant oxidation of Mn-derived states of MnPc, similar to the charge transfer as discussed in previous sections. This situation is nicely supported by model calculations of a MnPc/F_16_CoPc dimer [31,138]. The calculations demonstrate that the states of the two phthalocyanines combine to form new bonding and anti-bonding states. The Mn 3d*_xz_* and the Co 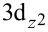 states hybridize and form a two-level system as illustrated qualitatively in Figure 15.

The occupation of the lower of these hybrid states also is related to the observed charge transfer. Intriguingly, as a result of our calculations the MnPc/F_16_CoPc dimer is characterized by a net spin of *S* = 2. Thus, the charge transfer is connected to a transfer/change of spin, which justifies to call the corresponding interface a spin-transfer interface with potential applications in the area of spintronics.

Finally, we note that also a bulk material consisting of MnPc/F_16_CoPc dimers could be prepared via the co-evaporation of these two materials. Spectroscopic studies of the resulting films confirmed the formation of MnPc/F_16_CoPc charge-transfer dimers in analogy to the related interface as discussed above [139]. The electronic excitation spectrum of these co-evaporated MnPc/F_16_CoPc films is characterized by a new feature at low energies (about 0.6 eV). Our density functional theory based calculations of the excitation spectrum reveal that this low-energy signal is due to transitions between the states of the dimer related two level system (see Figure 15).

## Conclusion

The compilation of our results on bulk compounds and interfaces based on manganese phthalocyanines and partners, where the phthalocyanine is either reduced or oxidized demonstrates the variability of MnPc in the formation of novel, potentially interesting systems. Moreover, apart from interesting electronic properties that are associated with the charge transfer in either case, the spin/magnetic state of MnPc must also be changed since Mn 3d orbitals participate in the charge transfer. Future studies will certainly unravel more details and intriguing features in these respects.
